# Increased Circulatory Interleukin-17A Levels in Patients with Progressive and Leukotrichial Vitiligo

**DOI:** 10.1155/2021/5524566

**Published:** 2021-04-21

**Authors:** Thai Van Thanh Le, Huy Ngoc Phan, Tran Ngoc Dang, Le Duy Pham

**Affiliations:** ^1^Department of Dermatology, University of Medicine and Pharmacy at Ho Chi Minh City, Ho Chi Minh City, Vietnam; ^2^Ho Chi Minh City Hospital of Dermato-Venereology, Ho Chi Minh City, Vietnam; ^3^Faculty of Public Health, University of Medicine and Pharmacy at Ho Chi Minh City, Ho Chi Minh City, Vietnam; ^4^Faculty of Medicine, University of Medicine and Pharmacy at Ho Chi Minh City, Ho Chi Minh City, Vietnam

## Abstract

**Background:**

Vitiligo is a chronic condition characterized by skin depigmentation. Although not life-threatening, it significantly impacts quality of life. The pathophysiology of vitiligo remains poorly understood, and treatment options are limited. Mounting evidence supports the importance of autoreactive T cells and, particularly interleukin-17A- (IL-17A-) secreting Th17 cells, in vitiligo. IL-17A targeting has been proven successful in various inflammatory dermatological conditions, including psoriasis and lupus erythematosus.

**Objective:**

We evaluated the relationship between serum levels of IL-17A and the clinicopathological characteristics of Vietnamese vitiligo patients.

**Methods:**

In this cross-sectional study, we analyzed data from 52 nonsegmental vitiligo patients and 50 age- and sex-matched healthy individuals. Serum levels of IL-17A were measured using an enzyme-linked immunosorbent assay. We evaluated the correlation between IL-17A levels and clinical characteristics including leukotrichia, disease duration, vitiligo activity, and body surface area involvement.

**Results:**

Patients with progressive vitiligo had significantly higher IL-17A levels than patients with stable vitiligo (*P* = 0.014) or healthy individuals (*P* = 0.002). In addition, serum IL-17A levels were higher in vitiligo patients with leukotrichia than in patients without it (*P* = 0.04). Furthermore, serum IL-17A levels were negatively correlated with age (*r* = −0.39, *P* = 0.004) and age of onset (*r* = −0.33, *P* = 0.016) in vitiligo patients.

**Conclusions:**

Higher serum levels of IL-17A in patients with progressive vitiligo and leukotrichia suggest a potential role of IL-17A in melanocyte destruction in the epidermis and the follicular matrix.

## 1. Introduction

Vitiligo is characterized by the progressive destruction of pigmentary cells in the epidermis and the hair follicles, which leads to white macules that are challenging to cure. The disease has an estimated prevalence of 0.1–2% of the world's population [1–3]. Although not life-threatening, vitiligo significantly impacts patients' quality of life, particularly those with facial lesions. The precise etiology of vitiligo remains unclear although (a) biochemical [4], (b) neural [5], and (c) autoimmune [6, 7] mechanisms seem to play a role in its onset. Notably, the immune-related mechanisms underlying nonsegmental vitiligo have been comprehensively investigated.

Changes in CD4+ T cell functions and the presence of autoreactive melanocyte-specific cytotoxic T cells play important roles in vitiligo pathogenesis [8, 9]. Importantly, the role of the CD8-CXCR3-CXCL9/10-IFN*γ* axis in the pathogenesis of nonsegmental vitiligo is becoming increasingly evident [10, 11]; however, targeting this pathway may lead to long-term adverse effects, such as skin cancer, as it plays a vital role in maintaining the cutaneous immunity [12]. On the contrary, T helper 17 (Th17) cells exert their biological functions by producing interleukin (IL)-17, which may exacerbate autoimmune inflammation in vitiligo. IL-17 also stimulates keratinocytes to produce several chemokines, resulting in recruitment of T cell, neutrophil, macrophage [13], and potential loss of melanocytes [14]. IL-17 is a potent stimulator of chemokine CCL20 production, which drives the migration of cytotoxic CD8+ T cells from systemic circulation into peripheral tissues [15, 16]. In mouse models, the migration of CD8+ T cells to the skin results in melanocyte loss [17, 18]. Additionally, IL-17 stimulates endothelial cells to express E- and P-selectins and the adhesion molecules ICAM-1 and VCAM-1, resulting in increased neutrophil migration [19].

Nevertheless, the role of IL-17A in vitiligo remains poorly understood. In this cross-sectional study, we evaluated the relationship between serum levels of IL-17A and the clinicopathological characteristics of Vietnamese individuals with nonsegmental vitiligo.

## 2. Materials and Methods

### 2.1. Study Subjects

We recruited 52 nonsegmental vitiligo patients (Group 1), including patients with focal acra-facial, mucosal, generalized, and universal vitiligo. Subjects who were treated with systemic or topical corticosteroids, or who received any other systemic immunosuppressive therapy in the preceding 2 months of recruitment, were excluded. Pregnant/lactating patients, or patients with a history of Hashimoto thyroiditis, Graves' disease, insulin-dependent diabetes, Addison's disease, alopecia areata, psoriasis, rheumatoid arthritis, or dysfunctional thyroid, were also excluded. We also enrolled 50 age- and sex-matched healthy individuals (Group 2) who did not have any criteria above as well as acute or chronic diseases. In Group 1, we recorded age, sex, duration of the disease, age of onset, family history, Koebner phenomenon, clinical features of the lesions, body area involvement (in quartile percentiles), and disease activity by using the vitiligo disease activity (VIDA) score [20]. The study was approved by IRB of University of Medicine and Pharmacy at Ho Chi Minh City (IRB number: 424/DHYD-HDDD).

### 2.2. Measurement of Serum IL-17A Levels by ELISA

Serum samples were collected from study subjects. Serum IL-17A levels were measured using an ELISA with a Human IL-17A ELISA Kit (ANOGEN Inc, Ontario, Canada) following the manufacturer's instructions.

### 2.3. Statistical Analysis

Data are expressed as mean ± standard deviation (SD), median and range, or prevalence. Mean values were compared using Mann–Whitney U (for two groups) or the Kruskal–Wallis test (for three or more groups). Spearman's rank correlation coefficient was used to investigate the relationship between clinical parameters. *P* values less than 0.05 were considered statistically significant. Statistical analyses were performed using R packages (RStudio Desktop, version 1.2 for Windows, RStudio Inc., Boston, USA).

## 3. Results

### 3.1. Clinical Characteristics of the Study Subjects

The clinical characteristics of the study subjects are shown in Table 1. Group 1 included 18 males (34.62%) and 34 females (65.38%), with a mean age of 32.71 ± 14.92 (range, 6–61 years). Group 2 included 18 males (36%) and 32 females (64%).

### 3.2. Serum Interleukin-17A Level in Patients and Controls

**Figure 1:**
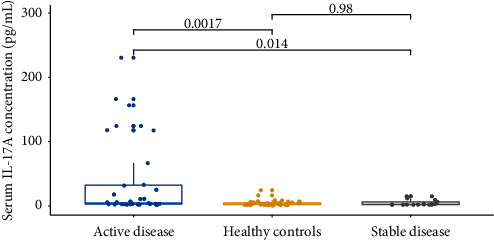
Serum levels of IL-17A in active and stable disease and healthy controls.

The serum IL-17A levels of Group 1 (median 3.09 (2.16–15.03) pgmL) were significantly higher than those of Group 2 (median 2.66 (1.86–4.80) pgmL) (*P* = 0.018). In addition, serum IL-17A levels of patients with progressive vitiligo (median 4.35 (2.78–32.26) pg/mL) were significantly higher than those with stable vitiligo (median 2.26 (1.94–6.32) pg/mL, *P* = 0.014) as well as healthy controls (median 2.66 (1.85–4.79) pg/mL, *P* = 0.002). However, there were no significant differences in serum IL-17A levels between the patients with stable disease and healthy controls (Figure 1).

### 3.3. Association of Serum IL-17A Levels with Clinical Characteristics

Vitiligo patients with leukotrichia had significantly higher serum levels of IL-17A than those without leukotrichia (8.27 (2.78–122.62) pg/mL vs 2.99 (2.02–10.38) pg/mL, *P* < 0.05). Nevertheless, serum IL-17A levels were not associated with sex, family history of vitiligo, affected body surface area, or lesional characteristics (including trichrome, confetti-like, perifollicular hyperpigmentation, Koebner phenomenon, and nevus halo) (Table 2).

**Figure 2:**
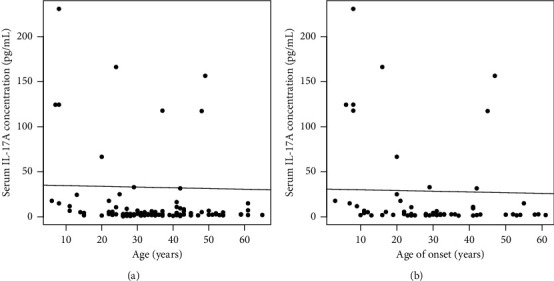
The correlations of serum IL-17A levels with age (a) and age of onset (b) in vitiligo patients.

In the subgroup analysis of active vitiligo, we also could not find the association between affected body surface area and the concentration of IL-17A (*r* = −0.15, *P* = 0.38). Furthermore, serum IL-17A levels were negatively correlated with age (*r* = −0.39, *P* = 0.004) and age of onset (*r* = −0.33, *P* = 0.016) in vitiligo patients but not in healthy controls (Table 3 and Figure 2).

## 4. Discussion

The pathophysiology of vitiligo remains understudied. Changes in humoral and cellular immunity are considered important causes of melanocyte destruction and subsequent vitiligo. Consistently, vitiligo is associated with autoimmune endocrinopathies [2]. Furthermore, the presence of autoantibodies against melanocyte antigens, including tyrosinase and tyrosinase-related protein (TYRP) 1 and 2, in the sera of vitiligo patients suggests an important role of humoral immunity in vitiligo pathophysiology [21]. In addition, high numbers of T-cell in the lesional margins in inflammatory vitiligo indicate the role of cellular immunity in vitiligo [22]. Besides the Th1 response and subsequent TNF-*α* and IFN-*γ* production, Th17 cells and L-17A have recently been implicated in the pathogenesis of vitiligo. IL-17 A targets multiple cell types, including fibroblasts, endothelial cells, epithelial cells, keratinocytes, and macrophages. Kotobuki et al. [23] investigated the biological effects of IL-17A and found that expression of MITF, an important transcriptional regulator of melanogenesis, and its downstream genes was reduced by more than 10% in melanocytes treated with IL-17. In addition, melanin production from IL-17-treated melanocytes decreased by approximately 30%, accompanied by profound morphological changes. In addition, IL-17 synergizes with various inflammatory mediators, including IL-1*α*, IL-6, and TNF-*α* [24], to inhibit melanocyte proliferation [25].

In this study, we found that serum IL-17A level was significantly increased in patients with nonsegmental vitiligo compared to healthy controls, confirming the findings of studies [26–30]. We also found that serum IL-17A levels were negatively correlated with age in patients with vitiligo but not in healthy controls. Previously, Kosar Hedayat et al. [31] demonstrated that vitiligo patients under 30 years had a lower quality of life than those aged above 30 years. Interestingly, mental stress is a trigger for vitiligo in 50–65% of vitiligo patients [32, 33], likely due to aggravation of innate and adaptive immunity [34]. Additionally, chronic stress polarizes immune response towards Th17 rather than Th2 response [35, 36]. Consequently, we hypothesized that the increased levels in younger vitiligo patients may be due to mental stress. However, further investigations are required to elucidate the relationship between IL-17A levels and age.

We also found a negative correlation between IL-17A levels and age of onset in vitiligo patients. This finding is in agreement with the findings of Basak et al. [25] but contradict those of Zhou et al. [37]. This suggests that IL-17A may contribute to the immune response in early-onset vitiligo. In addition, Arcos-Burgos et al. [38] demonstrated an association of early onset vitiligo with HLA-DR4, which is involved in CD4+ T cell activation and subsequent production of proinflammatory cytokines, including IL-17 and IFN-*γ* [39]. More studies are needed to understand the role of IL-17A in early onset vitiligo.

A correlation between serum IL-17A levels and body surface involvement has been reported in previous studies [25, 26, 29]. However, we found no significant association between these two factors, consistent with Tembhre et al. [28] and Zhou et al. [37]. Consistent with previous findings [28, 37, 40, 41], we found that IL-17A levels were correlated with progressive vitiligo, suggesting that IL-17A might be involved in vitiligo induction and progression. Zhen et al. [41] reported that while serum IL-17A levels were significant higher in the patients with nonsegmental vitiligo, there were no significant differences in serum levels of IFN-*γ*, IL-4, and TGF-*β*1. Furthermore, in this study, the numbers of peripheral CD4^+^IL-17A^+^ Th17 cells were significantly higher in vitiligo patients compared to healthy volunteers; however, no differences were observed in the numbers of peripheral CD4^+^IL-4^+^ Th2 or regulatory T cells. These findings suggest that IL-17 may be crucial in the onset and progression of autoimmune nonsegmental vitiligo. However, regarding the biomarkers of disease activity, circulating cytokines (IL-1*β*, IL-17, IFN-*γ*, and TGF-*β*), autoantibodies, oxidative stress markers, soluble CDs (sCD25 and sCD27), and chemokines (CXCL9 and CXCL10) remain competing [42].

Follicular melanocytes serve as a melanocyte reservoir, facilitating repigmentation in vitiligo. The presence of leukotrichia in white patches may be a sign of refractory vitiligo [43], and untreated leukotrichia could severely impact the quality of life [44]. The understanding of follicular vitiligo pathogenesis and the relationship between IL-17A and leukotrichia, in particular, is limited. In the present study, we found that nonsegmental vitiligo patients with leukotrichia had significantly higher serum levels of IL-17A than patients without leukotrichia. Gan et al. [45] reported the presence of perifollicular lymphocytes in the infundibular region of the hair follicles, while melanocytes were absent in the basal layer of the epidermis and the hair follicle. These findings suggest that both CD8+ and CD4+ T cells could attack hair follicle melanocytes [46]. In addition, Rongioletti et al. [47] reported a repigmentation of follicular melanocytes in psoriasis patients after treatment with secukinumab, supporting the role of IL-17A in the pathogenesis of follicular melanocyte destruction. Further studies are warranted to understand the role of IL-17 in the pathogenesis of vitiligo.

This study had some limitations that need to be addressed. First of all, the concentration of IL-17A was only measured in the serum, but not in the skin or follicles of the vitiligo patients. Thus, further researches implemented at the lesional sites should be conducted. Secondly, the size sample of our study was modest partly due to the uncommon characteristic of the vitiligo. Finally, this was a cross-sectional study in which a causal relationship could not be established.

## 5. Conclusions

In conclusion, this study provides evidence of that IL-17A may play a crucial role in the induction of vitiligo along with other factors, namely, the genetics, environment, and inflammatory cytokines. In addition, we found that serum levels of IL-17A were significantly associated with leukotrichia, suggesting the role of IL-17A in the destruction of follicular melanocytes.

## Figures and Tables

**Table 1:** Clinical characteristics of study subjects.

	Group 1 (*n* = 52)	Group 2 (*n* = 50)	*P* value
Age (mean ± SD)	32.71 ± 14.92	36.34 ± 10.34	0.22

Male, *n* (%)	18 (34.62%)	18(36%)	0.88

Age of onset, *n* (%)			
≤30 years	29 (55.77%)	NA	NA
>30 years	23 (44.23%)		

Duration of disease, *n* (%)			
<5 years	40 (76.92%)	NA	NA
5–10 years	8 (15.39%)		
>10 years	4 (7.69%)		

Family history of vitiligo, *n* (%)	11 (21.15%)	NA	NA

Affected body surface area, *n* (%)			
<3%	32 (61.54%)	NA	NA
3–10%	13 (25.00%)		
>10%	7 (13.46%)		

Triggers, *n* (%)			
Trauma	11 (21.15%)	NA	NA
Psychological stress	19 (36.54%)		
Sunburn	6 (11.54%)		
Pregnancy	6 (11.54%)		

Vitiligo activity, *n* (%)		NA	NA
Progressive			
VIDA +4	23 (44.23%)		
VIDA +3	12 (23.08%)		
Stable			
VIDA +2	7 (13.46%)		
VIDA +1	8 (15.39%)		
VIDA 0	0 (0.00%)		
VIDA −1	2 (3.84%)		

Clinical variants, *n* (%)		NA	NA
Focal	11 (21.15%)		
Acro/acrofacial	4 (7.69%)		
Mucosa	1 (1.93%)		
Generalized	36 (69.23%)		
Universal	0 (0.00%)		

Lesion characteristics, *n* (%)		NA	NA
Trichrome	31 (59.62%)		
Confetti-like	10 (19.23%)		
Perifollicular hyperpigmentation	22 (42.31%)		
Leukotrichia	14 (26.92%)		
Koebner phenomenon	12 (23.08%)		
Nevus halo	2 (3.85%)		

NA, not available.

**Table 2:** The association of serum IL-17A levels with clinical characteristics of vitiligo patients.

Clinical characteristics	*N*	Serum IL-17 level (pg/mL)	*P* value
Gender			
Females	34	3.12 (2.16–15.03)	0.81
Males	18	2.78 (2.11–10.08)	

Ages			
≤30 years	21	10.68 (3.09–32.91)	**0.003**
>30 years	31	2.78 (1.96–5.34)	

Vitiligo activity			
Stable	17	2.26 (1.94–6.32)	**0.013**
Progressive	35	4.35 (2.78–32.26)	

Family history of vitiligo			
Yes	11	2.26 (1.96–49.10)	0.69
No	41	3.09 (2.47–11.86)	

Leukotrichia			
Yes	14	8.27 (2.78–122.62)	**0.04**
No	38	2.99 (2.02–10.38)	

Affected body surface area			
<3%	32	3.08 (2.24–12.02)	0.82
3–10%	13	4.35 (2.42–11.86)	
>10%	7	2.78 (1.78–16.	

*P* values were obtained by the Mann–Whitney U test. Data were shown as median (25th–75th).

**Table 3:** The correlations of serum IL-17A levels with ages and age of onset in the study groups.

	Group 1 (*n* = 52)	Group 2 (*n* = 50)
Age	*r* = −0.39, *P* = 0.004	*r* = 0.27, *P* = 0.06
Age of onset	*r* = −0.33, *P* = 0.016	NA

NA, not available.

## Data Availability

Due to privacy and ethical concerns, neither the data nor the source of the data can be made available.
